# Iris reconstruction using autologous iris preserved in cold balanced salt solution for 8 hours in iatrogenic total iridodialysis during cataract surgery: a case report

**DOI:** 10.1186/s12886-017-0432-4

**Published:** 2017-04-04

**Authors:** Seung Pil Bang, Jong Hwa Jun

**Affiliations:** Department of Ophthalmology, Keimyung University School of Medicine, #56, Dalseong-ro, Jung-gu, Daegu, 700-712 Korea

**Keywords:** Balanced salt solution, Iatrogenic, Iris reconstruction, Total iridodialysis

## Abstract

**Background:**

A large iris defect or extensive iridodialysis can be an intractable cause of visual disturbance, photophobia, glare, monocular diplopia, or cosmetic deformity. The implantation of an artificial iris substitute could be an effective option, but this can cause a reduction in endothelial cell density. We succeeded in the anatomical restoration of iris tissue that was totally dialyzed out of the eye, and was preserved in cold balanced salt solution for 8 h. Engrafted iris tissue was maintained within the aqueous humor.

**Case presentation:**

A 71-year-old man was referred to our clinic for management of an iatrogenic total iridodialysis. The totally dialyzed iris tissue was immediately preserved in sterile cold balanced salt solution and packed in a sterile biopsy bottle that was surrounded with ice cubes. Under general anesthesia, a pars plana vitrectomy was performed to remove the remaining lens cortex and vitreous fiber anterior to the equator. A sulcus-positioned intraocular lens (IOL) was repositioned and fixed by *ab externo* scleral sutures. Preserved iris tissue was inserted and ironed using both iris spatula and ocular viscoelastic devices. Five-point *ab interno* scleral sutures were made 1.0 mm posterior to the limbus.

**Conclusions:**

The engrafted iris was successfully maintained for 6 months and did not undergo any atrophic change or depigmentation, which may be caused by primary implantation failure due to a blocked blood supply.

## Background

The iris functions as a light-limiting diaphragm. Iris defects, whether traumatic or iatrogenic, can cause deterioration in visual acuity, photophobia, glare, as well as diplopia if the edge of the phakic or pseudophakic lens is involved [1]. In addition, an extensive defect can be a significant cosmetic concern [2]. Various techniques to overcome partial or total iris defects have been described, including iridoplasty, coloured contact lenses, and corneal tattooing [3]. Implantation of an artificial iris substitute is a new and effective therapeutic option, but can cause significant reduction of endothelial cell density or even corneal decompensation after surgery [4]. Thus, a widespread defect could be a vision-threatening situation. We encountered a case of iatrogenic total iridodialysis during cataract surgery. We restored the structure of the autogenous iris, which had been preserved in cold balanced salt solution for 8 h.

## Case presentation

**Fig. 1:**
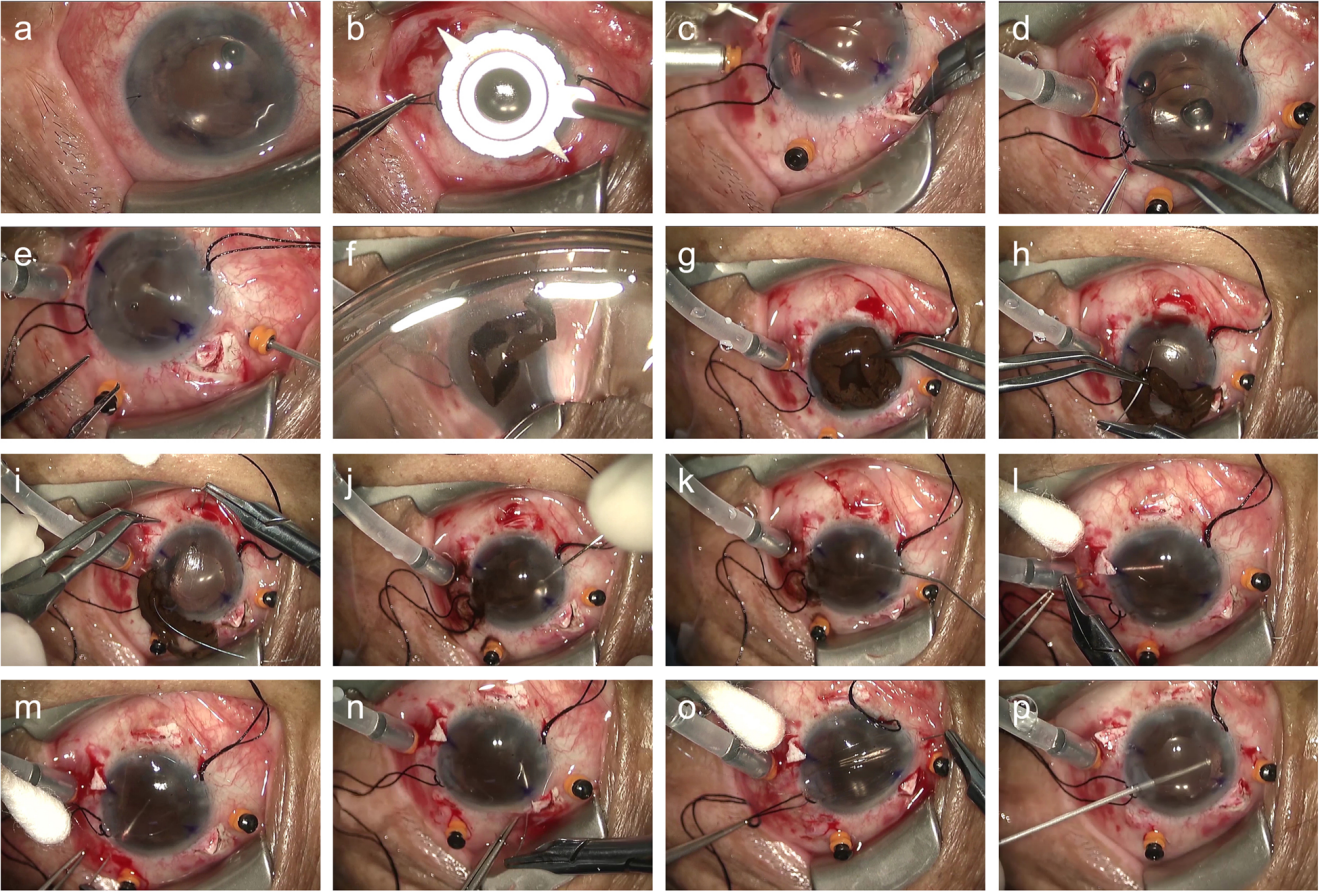
Intraoperative photographs of the iridopexy of an autologous iris in iatrogenic total iridodialysis. **a** A complete iris defect with remaining lens cortex, a ruptured posterior capsule of the lens with a radial tear and an intraocular lens (IOL) implanted in the sulcus position were observed. **b** A toric axis marker was used to indicate the fixation axis. **c** After scleral flaps were prepared in 2 positions 180° apart, a 10–0 Prolene suture was passed through the bed of half-thickness scleral flaps 2.0 mm posterior to the limbus. **d** Implanted IOL was repositioned using transscleral fixation using an *ab externo* method. **e** Pars plana vitrectomy was performed to remove the remaining lens cortex material and vitreous fiber anterior to the equator. **f** The transferred iris was examined and showed no signs of necrosis. **g** The iris was spread out; the wider part of the iris was located inferiorly. **h**, **i** A 10–0 Prolene suture was consecutively passed through the iris and sclera. **j**, **k** Using an iris spatula and ocular viscoelastic devices (OVDs), the iris was inserted into the anterior chamber completely. **l**-**o** Four more iridopexies were performed. **p** The remaining vitreous, OVDs, and dispersed iris pigments were removed using a vitreous cutter

A 71-year-old man was referred to our clinic for treatment of an iatrogenic total iridodialysis. Just before the referral, his iris had been totally torn out and jammed into the hinge of a prechopper during the removal of an instrument during cataract surgery. Examination revealed a visual acuity (VA) of hand-motion in the left eye. A complete iris defect with remaining lens cortex, a ruptured posterior lens capsule with radial tear of the capsule, and an intraocular lens (IOL) implanted in the sulcus were noted (Fig. 1a). The totally dialyzed iris was sent to our clinic preserved in sterile cold balanced salt solution, packed in a sterile biopsy bottle surrounded by a towel to prevent direct contact with ice cubes, and was transported in an icebox.

We decided to perform surgery under general anesthesia considering the patient’s poor cooperation due to dementia. To minimize IOL decentration during scleral fixation, we used a toric axis marker and marked the fixation axis (Fig. 1b). After the scleral flaps were in two positions 180° apart, a 10–0 polypropylene suture was passed through the bed of half-thickness scleral flaps 2.0 mm posterior to the limbus (Fig. 1c). A sulcus positioned IOL (PC-60 AD, HOYA Corporation, Tokyo, Japan) was repositioned and fixed by *ab externo* scleral sutures (Fig. 1d). We conducted a pars plana vitrectomy to remove the remaining lens cortex material and vitreous fibre anterior to the equator to avoid trapping the vitreous during the iris-fixating suturing (Fig. 1e). The preserved iris was examined. It did not show any signs of necrosis but kept its own color and morphology soundly (Fig. 1f). We spread out the iris on the patient’s cornea to estimate the range of damage and locate a wider part of the iris inferiorly to minimize the glare after iridopexy (Fig. 1g). A 10–0 Prolene on a CIF4 needle (Ethicon, Somerville, New Jersey, USA) was consecutively passed through the iris (Fig. 1h) and sclera 1.0 mm posterior to the limbus at the 6’ O/C position (Fig. 1i). Properly using both an iris spatula and ocular viscoelastic devices (OVDs), we inserted the iris into the anterior chamber completely and unfolded it to its proper position (Fig. 1j, k). The estimated cool-to-anterior chamber insertion time of the preserved iris was 8 h. Four more points of *ab interno* scleral sutures (4’, 1:30, 10:30 and 8’ O/C positions in sequence) were made (Fig. 1l-o). Then, the remaining vitreous, OVDs, and dispersed iris pigments were removed using a vitreous cutter (Fig. 1p).

**Fig. 2:**
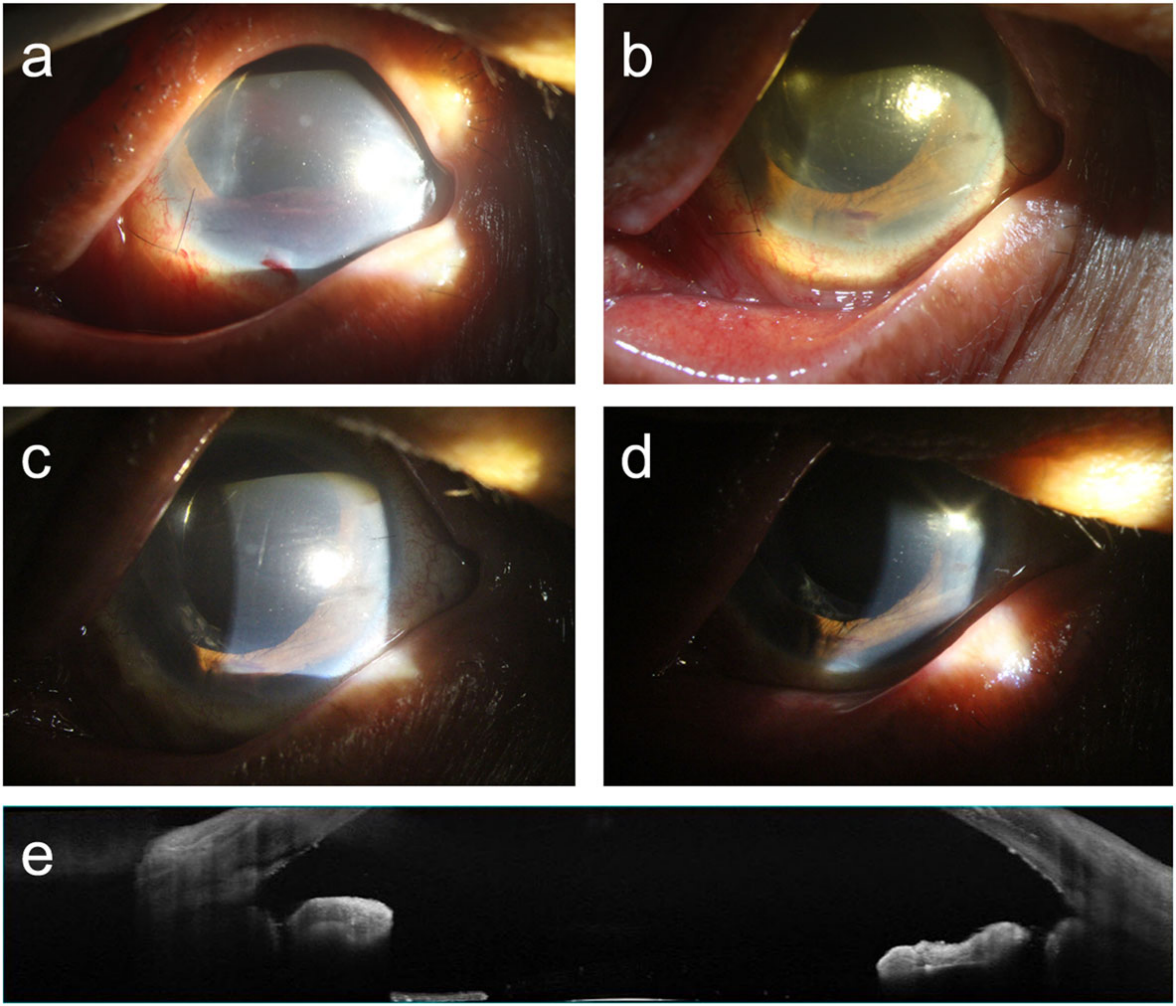
Postoperative slit-lamp examination and tomographic image. **a** Bleeding from the torn root of the iris at 1 week postoperatively. **b** Hyphema decreased at 3 weeks postoperatively. **c**, **d** No atrophy or pigment loss and no signs of inflammation at 4 and 7 weeks postoperatively. **e**. Anterior optical coherence tomographic image showed morphologic stability of the iris

One week postoperatively, intraocular pressure (IOP) increased up to 30 mmHg because of hyphema from the torn root of the iris (Fig. 2a); however, 3 weeks postoperatively, hyphema decreased with improved VA (20/200) and lowered IOP (15 mmHg) (Fig. 2b). At 4 weeks postoperatively, a much improved VA (20/100) and lowered IOP (14 mmHg) were detected (Fig. 2c). At 7 weeks postoperatively, VA was 20/63, IOP was 14 mmHg and there were no signs of inflammation in the anterior chamber (Fig. 2d). Until 6 months postoperatively, the engrafted iris did not have any signs of atrophic change, depigmentation, or inflammation; the patient complained of minimal glare, and the uncorrected VA was 20/25 with the IOP of 13 mmHg (Fig. 2e).

## Discussion

An intact iris diaphragm is essential for accurate visual function as it decreases the aberrations arising from the crystalline lens and increases the depth of focus [3]. Coloured contact lenses may not be acceptable or tolerated. Corneal tattooing leaves a permanent opacity in the cornea, and the results are unpredictable. In the absence of readily available iris prosthetic devices in many areas of the world, including the USA, an approach must be tailored appropriately for the surgical challenge. For that reason, we decided to perform a remodeling of the autologous iris. Reconstruction of a totally dialyzed iris was a technical challenge because the forces applied by the sutures often leave the iris and pupil with an irregular and distorted appearance. To the best of our knowledge, this is the first report of restoration of an autologous iris in iatrogenic total iridodialysis. Iris tissue defects can also be cosmetically upsetting for the patient, and an aesthetically pleasing surgical outcome is often a key concern. Fortunately, our patient expressed satisfaction with the cosmetic appearance of his left eye.

We were concerned that even if we could do the reconstruction successfully, a primary implantation failure with atrophic change or depigmentation of the engrafted iris and severe inflammation of the anterior chamber would be inevitable. However, the iris maintained its own morphological stability without a direct blood supply from ciliary vessels for 6 months postoperatively; conceivably, it might have been sustained through the nutrition supply from the aqueous by principle of diffusion. Additionally, the engrafted iris might have restored its vasculature near its remnant root after implantation. One animal study showed that isolated acapsular glomeruli transplanted into the anterior chamber of the mouse eye were capable of spontaneously regaining access to the recipient vasculature and retaining their structure and function [5], indicating the possibility of regrowth of the engrafted iris vessels in our case.

Though cases have been reported in the literature of traumatic iridodialysis following blunt injury to pseudophakic eyes [6–12], our case is unique in that it is the first case of iatrogenic iridodialysis during cataract surgery instead of trauma [13]; the iris reconstruction was also unique in using an autologous iris instead of prosthetic iris implantation [1, 3, 14]. Considering the short follow-up period of this case, long-term observation is planned to exclude the possibility of chronic atrophic change or depigmentation of the engrafted iris. It might also be necessary to conduct studies on the engrafted iris vasculature such as fluorescein angiography [15, 16], indocyanine green angiography [17] or optical coherence tomography angiography [18].

## Conclusions

We successfully performed the reconstruction of a totally avulsed iris during cataract surgery and obtained cosmetically favourable morphology of the engrafted iris. Despite 8 h of extracorporeal preservation of the iris and depletion of a direct blood supply after reconstruction, the implanted iris maintained its own stability without any complication or graft failure.

## Abbreviations

IOLs: Intraocular lens
IOP: Intraocular pressure
OVDs: Ocular viscoelastic devices
VA: Visual acuity

## References

[1] Burk SE, Da Mata AP, Snyder ME, Cionni RJ, Cohen JS, Osher RH (2001). Prosthetic iris implantation for congenital, traumatic, or functional iris deficiencies. J Cataract Refract Surg.

[2] Blackmon DM, Lambert SR (2003). Congenital iris coloboma repair using a modified McCannel suture technique. Am J Ophthalmol.

[3] Hanumanthu S, Webb LA (2003). Management of traumatic aniridia and aphakia with an iris reconstruction implant. J Cataract Refract Surg.

[4] Mayer CS, Reznicek L, Hoffmann AE (2016). Pupillary reconstruction and outcome after artificial iris implantation. Ophthalmology.

[5] Kistler AD, Caicedo A, Abdulreda MH, Faul C, Kerjaschki D, Berggren PO, Reiser J, Fornoni A (2014). In vivo imaging of kidney glomeruli transplanted into the anterior chamber of the mouse eye. Sci Rep.

[6] Navon SE (1997). Expulsive iridodialysis: an isolated injury after phacoemulsification. J Cataract Refract Surg.

[7] Ball J, Caesar R, Choudhuri D (2002). Mystery of the vanishing iris. J Cataract Refract Surg.

[8] Walker NJ, Foster A, Apel AJ (2004). Traumatic expulsive iridodialysis after small-incision sutureless cataract surgery. J Cataract Refract Surg.

[9] Sullivan CA, Murray A, McDonnel P (2004). The long-term results of nonexpulsive total iridodialysis: an isolated injury after phacoemulsification. Eye (Lond).

[10] Kahook MY, May MJ (2005). Traumatic total iridectomy after clear corneal cataract extraction. J Cataract Refract Surg.

[11] Muzaffar W, O’Duffy D (2006). Traumatic aniridia in a pseudophakic eye. J Cataract Refract Surg.

[12] Eom Y, Kang SY, Song JS, Kim HM (2013). Traumatic aniridia through opposite clear corneal incision in a pseudophakic eye. J Cataract Refract Surg.

[13] Jovanovic M, Radosavljevic P (1991). Reconstruction of the iris in iridodialysis after a contusion injury of the eye. Srp Arh Celok Lek.

[14] Ozturk F, Osher RH, Osher JM (2006). Secondary prosthetic iris implantation following traumatic total aniridia and pseudophakia. J Cataract Refract Surg.

[15] Brancato R, Bandello F, Lattanzio R (1997). Iris fluorescein angiography in clinical practice. Surv Ophthalmol.

[16] Craandijk A, Aan de Kerk AL (1970). Fluorescence angiography of the iris. Br J Ophthalmol.

[17] Goto T, Shimura M, Nakazawa M (1999). Indocyanine green iris angiography of lung carcinoma metastatic to the iris. Graefes Arch Clin Exp Ophthalmol.

[18] Skalet AH, Li Y, Lu CD, Jia Y, Lee B, Husvogt L, Maier A, Fujimoto JG, Thomas Jr CR, Huang D. Optical coherence tomography angiography characteristics of iris melanocytic tumors. Ophthalmology. 2017;124(2):197–204. doi: 10.1016/j.ophtha.2016.10.003. Epub 2016 Nov 14. 10.1016/j.ophtha.2016.10.003 PMC5272860 27856029

